# Implementation of an organizational intervention to improve low-wage food service workers’ safety, health and wellbeing: findings from the Workplace Organizational Health Study

**DOI:** 10.1186/s12889-021-11937-9

**Published:** 2021-10-16

**Authors:** Glorian Sorensen, Susan E. Peters, Karina Nielsen, Elisabeth Stelson, Lorraine M. Wallace, Lisa Burke, Eve M. Nagler, Hamid Roodbari, Melissa Karapanos, Gregory R. Wagner

**Affiliations:** 1 Dana-Farber Cancer Institute, 450 Brookline Ave, Boston, MA 02215 USA; 2 Harvard T.H. Chan School of Public Health, 677 Huntington Ave, Boston, MA 02115 USA; 3 University of Sheffield, Conduit Rd, Sheffield, S10 1FL UK

**Keywords:** Organizational interventions, Occupational health and safety interventions, Process evaluation, Intervention implementation, Total worker health, Food service workers, Participatory intervention, Low wage workers, Healthy work design

## Abstract

**Background:**

Many organizational interventions aim to improve working conditions to promote and protect worker safety, health, and well-being. The Workplace Organizational Health Study used process evaluation to examine factors influencing implementation of an organizational intervention. This paper examines the extent to which the intervention was implemented as planned, the dose of intervention implemented, and ways the organizational context hindered or facilitated the implementation of the intervention.

**Methods:**

This proof-of-concept trial was conducted with a large, multinational company that provides food service through contractual arrangements with corporate clients. The 13-month intervention was launched in five intervention sites in October 2018. We report findings on intervention implementation based on process tracking and qualitative data. Qualitative data from 25 post-intervention interviews and 89 process tracking documents were coded and thematically analyzed.

**Results:**

Over the 13-month intervention, research team representatives met with site managers monthly to provide consultation and technical assistance on safety and ergonomics, work intensity, and job enrichment. Approximately two-thirds of the planned in-person or phone contacts occurred. We tailored the intervention to each site as we learned more about context, work demands, and relationships. The research team additionally met regularly with senior leadership and district managers, who provided corporate resources and guidance. By assessing the context of the food service setting in which the intervention was situated, we explored factors hindering and facilitating the implementation of the intervention. The financial pressures, competing priorities and the fast-paced work environment placed constraints on site managers’ availability and limited the full implementation of the intervention.

**Conclusions:**

Despite strong support from corporate senior leadership, we encountered barriers in the implementation of the planned intervention at the worksite and district levels. These included financial demands that drove work intensity; turnover of site and district managers disrupting continuity in the implementation of the intervention; and staffing constraints that further increased the work load and pace. Findings underscore the need for ongoing commitment and support from both the parent employer and the host client.

**Trial registration:**

This study was retrospectively registered with the Clinical Trials. Gov Protocol and Results System on June 2, 2021 with assigned registration number NCT04913168.

## Background

A growing literature emphasizes the importance to worker safety, health, and well-being of organizational interventions that improve working conditions by changing the design, organization and management of work [1–6]. Despite some inconsistencies across studies [7], research has demonstrated that organizational interventions to increase decision latitude, reduce work intensity, support teams, or build leadership have contributed to improved well-being [8–10], psychological health [10–14], and reduced sickness absence [11, 14–16]. Inconsistencies in the effectiveness of these interventions may reflect insufficient implementation of the intervention as well as inadequate fit with the specific setting in which the intervention is implemented [1, 17–19].

The Workplace Organizational Health Study tested an intervention to improve the work organization as a means of promoting and protecting the safety, health, and well-being of low-wage food service workers. The study was conducted in collaboration with a food service organization (the parent employer) that contracted with client companies (the hosts) to provide staffing for the hosts’ in-house cafeterias [20]. Food service workers regularly encounter physically demanding work; job insecurity; uncertainty around work hours, contributing to instability in earnings; repetitive work; and low job decision latitude and autonomy [21–24]. The contracted nature of these workplaces blurs accountability for workers’ health and safety, thereby potentially compounding risks to workers [25]. Increasingly, low-wage workers are employed under contractual work arrangements such as these [26]. Given their potential impact, organizational interventions may be of particular importance for these low-wage workers [27, 28]. Furthermore, understanding the process of implementing interventions in these settings has important implications for organizational interventions in similar settings.

Process evaluation provides a method for assessing the implementation of interventions, allowing researchers to determine the extent to which interventions are delivered as planned and to identify barriers and facilitators encountered [1, 29–31]. These valuable insights into implementing interventions contribute to future interventions and related research [32]. These methods have been applied broadly in workplace studies to understand the process of intervention implementation [33–40]. In this study, the intervention was guided by our prior research [41, 42] and formative research of contextual factors of this setting [43]. Using qualitative and quantitative methods, this paper addresses two key questions related to the implementation of this organizational intervention: (1) Intervention Implementation: To what extent was the intervention implemented as planned (fidelity) and what was the “dose” of intervention implemented? (2) Context and process: To what extent did the organizational and broader contexts hinder or facilitate the process of implementing the intervention? This analysis provides insights on the process of implementing an organizational intervention within a low-wage food service setting, and on the challenges faced in implementing this intervention in a complex, multi-level organization that includes both the parent employer and host organizations.

## Methods

### Study design

The Workplace Organizational Health Study was a proof-of-concept trial designed to test our a priori central hypothesis that a multi-level participatory intervention targeting the work organization and environment could be feasibly implemented and would show promising improvements in worker health, safety, and well-being [20]. This paper focuses on the implementation of the intervention based on process evaluation and post-intervention interviews with site managers and leadership.

**Fig. 1:**
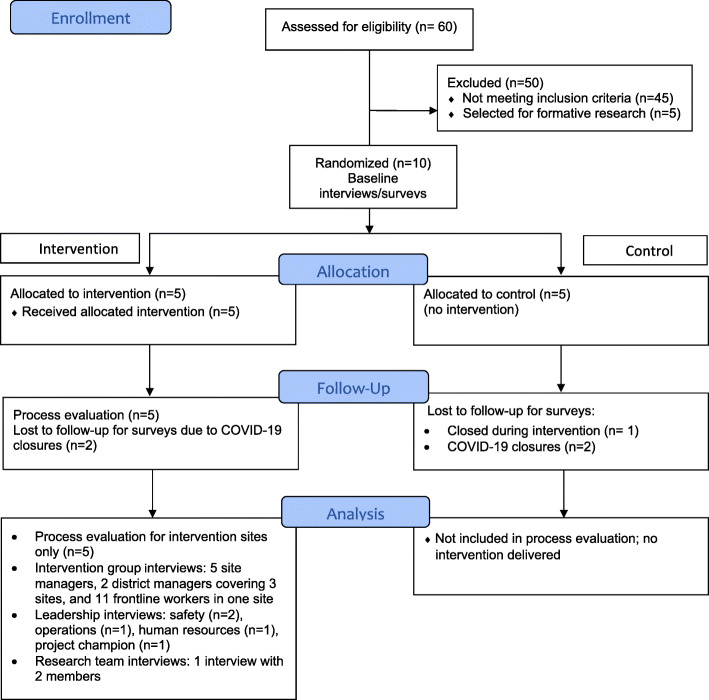
Study Design

This study was conducted in collaboration with a large, multinational company that provides food service through contractual arrangements with corporate clients. The participating worksites were located in corporate settings in the Greater Boston area of Massachusetts (US). Ten worksites were selected from 60 eligible worksites in the geographical region to participate in this cluster randomized trial. Following completion of baseline assessments, worksites were blocked on size (fewer than 15 employees versus 15 or more employees) and randomly assigned to each condition (see Fig. 1). The 13-month intervention was launched in five intervention sites in October 2018. One control site was closed prior to the end of the study, leaving five intervention sites and four control sites. Post-intervention data were collected from mid-January 2020 until early March 2020. Due to increasing restrictions related to the COVID-19 pandemic, final data collection could not be completed in four sites, thus leaving only five of the original ten sites in the study. This paper uses qualitative and quantitative process evaluation data collected during implementation of the intervention and data from qualitative interviews collected at the end of the intervention.

This study was approved by the Harvard T.H. Chan School of Public Health Institutional Review Board (Protocol # IRB16–0488). Participants provided informed consent prior to participating in any data collection.

### Setting

Organizational interventions focused on improving organizational and psychosocial conditions of work have been recognized as an important avenue for impacting workers’ safety, health and well-being. In the United States as well as elsewhere, such as in the European Union, Australia, and Canada, employers have obligations to ensure employees’ health and safety by addressing workplace hazards–including those that harm both physical and mental health–through legislations and occupational health and safety standards [44–47].

The food service sector accounts for approximately 4.5 million jobs in the U.S., and is expected to grow 17% between 2020 and 2030–a much higher growth rate than the average for all occupations [48]. U.S. food service workers also have higher rates of injuries than the average of all other industries, [49] and are exposed to a hazardous physical and psychosocial work environment. Beyond the U.S., many other countries also employ low wage workers in the food service sector, who also experience high rates of injuries and are exposed to similar hazards in their work environment that can have considerable impacts on their safety, health, and wellbeing. For example, 59% of workers in the European hospitality sector report poor quality of work, in terms of low skills and discretion, discrimination and career prospects [50].

### Study population and sample

Eligible worksites employed between 7 and 30 employees at baseline; were located in the Greater Boston area; had contracts with their corporate clients that were expected to last through the intervention period; did not have security issues limiting investigator access to the worksite; agreed to the planned data collection efforts; and agreed to be randomly assigned to the intervention or control condition following baseline data collection. For the purpose of this manuscript focusing on the process of implementing the intervention in sites assigned to the intervention, we focused on the intervention group only. Although the research team provided informational materials for site managers to use in introducing the study to their corporate clients, clients were not directly engaged in the implementation of the intervention.

The research team engaged managers across multiple organizational levels in the intervention and evaluation. *Site managers* were responsible for operations and supervised frontline workers at each worksite. The five site managers at intervention worksites were central to all phases of the intervention planning and implementation. At the *leadership level,* district managers provided direct supervision of site managers; additionally, senior leadership representatives from human resources, health and safety, and operations contributed to planning and implementing district-level policies and practices. At the national level, the research team collaborated with a project champion who provided corporate-level support for the study. In addition, *frontline workers,* including chefs, cooks, food preparers, servers, dishwashers, and cashiers, were surveyed at baseline and post-intervention; due to closures related to the COVID-19 pandemic, post-intervention survey data collection could not be completed.

### Intervention

Based on guidelines for implementing a Total Worker Health intervention, the intervention was designed to improve three working conditions: safety and ergonomics, work intensity, and job enrichment [51]. These working conditions were identified as priorities based on formative research conducted in five worksites that were part of the same company but not part of the proof-of-concept trial [43]. Four essential elements (leadership commitment, participation, communication, and tailoring for fit), defined as mechanisms promoting the intervention’s intended effects, guided planning and informed intervention implementation. The intervention focused on two levels: (1) the worksite level, including site managers as well as frontline workers engaged in collaboration with site managers, and (2) the leadership level, including district managers, senior leaders whose functions were central to the targeted working conditions (e.g., human resources, health and safety, and operations), and the project champion, who represented national leadership [52].

At the worksite-level, the research team provided consultation, tools, and technical support to site managers. A research team member was assigned to be the primary representative of the project within each worksite, with responsibility for working with site managers to implement the intervention related to each of the three sequential modules. For each module, the intervention included (1) site-specific assessments of current working conditions – including potential challenges, opportunities for improvement, and available tools and resources – which generated a report and recommendations for each module; (2) tools for developing and implementing an action plan to address these recommendations; (3) consultation and technical support to implement the recommended solutions for improving the targeted working conditions, provided through in-person and phone meetings between the research team representative and individual site managers, including guidance on participatory approaches to engage frontline workers in the change process; (4) tools for engaging frontline workers in the targeted changes; and (5) training and group discussions for site managers to facilitate changes in the Work Intensity and Job Enrichment modules.

At the leadership level, prior to initiating the intervention, the research team reviewed the intervention plans with senior leadership and district-level managers to ensure their commitment to the project. The research team periodically met with leadership representatives to identify relevant existing resources, policies and programs that could support site-specific interventions; address barriers and challenges to intervention implementation; and identify corporate-wide tools and resources that might align with the intervention and could be adapted to support the targeted organizational changes. Meetings with senior and district leadership also provided an opportunity for dialog, with the aim of creating feedback loops between the leadership and site levels throughout planning and implementation of the intervention. These meetings were conducted in parallel with the sequential modules.

**Fig. 2:**
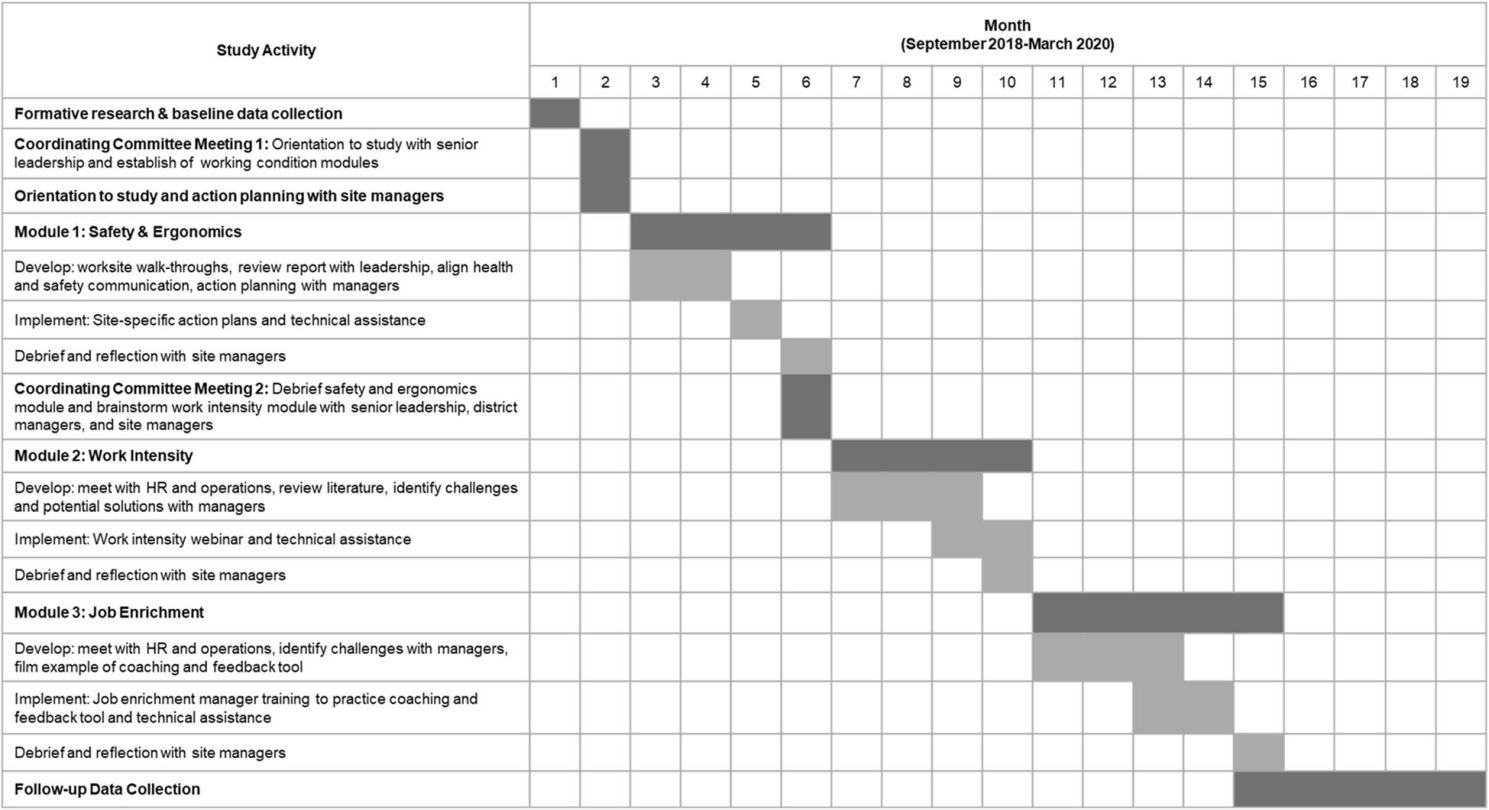
Timeline of intervention activities

The intervention focused on three sequentially-delivered modules aligned with the targeted working conditions (Fig. 2). Implementation of each module was guided by objectives and standardized protocols and used materials and tools to implement the intervention related to each site’s identified areas for improvement within each of the three working conditions. The intervention was structured so that all sites would address the same three working conditions, although the objectives were tailored to each site based on the site-specific assessments. The modules included the following:

1. Safety and Ergonomics (e.g., equipment use; slips and falls; prolonged standing; lifting and carrying demands). The Safety and Ergonomics assessment used a walkthrough and interview, conducted by an industrial hygienist on our research team, to identify site-specific areas of improvement [53]. The report informed priority setting for the action planning process, including development of strategies for prioritizing actions to address areas for improvement, ways to engage employees in the process, and identification of needed resources. At the district management and senior leadership levels, the research team shared an aggregate report of walkthrough results for their review and further identification of tools and resources to address the areas needing improvement.
2. Work Intensity (the pace of work and demands placed on both managers and employees). Work intensity was often exacerbated by insufficient staffing, unexpected catering obligations, and competing demands on the site manager’s time. The assessment helped to determine priorities for each site. The research team worked with the site managers to identify steps to address these priorities, brainstorm strategies for encouraging frontline workers’ input on solutions and implementation, and identify resources and tools to support each site’s goals. After a review with site managers, members of the research team discussed issues contributing to work intensity with senior leaders and district managers to consider opportunities to address root causes. In a group phone discussion, the research team reviewed the Work Intensity aggregate report with site managers to brainstorm best practice solutions that could be applicable to their accounts.
3. Job Enrichment (providing feedback and coaching to employees and offering opportunities for career advancement). This module promoted site managers’ use of an existing corporate tool for providing coaching and feedback to employees. The organization’s Human Resources leadership identified this resource – regularly used to support productive coaching and feedback at the management level and above – and provided input as the research team customized the tool for use with frontline workers. A webinar introduced an adapted coaching and feedback tool to site managers to help them communicate with frontline workers to identify opportunities for performance improvement and career growth on a regular basis.

Throughout the study, the research team also provided guidance and technical assistance to managers on how to facilitate a participatory process, focusing specifically on ways to engage their employees within a demanding food service setting. The research team developed scripts for huddles (short team meetings) for each working condition and reviewed them with site managers. Site managers utilized the script to facilitate a huddle (or multiple huddles) with frontline workers to discuss each of the working conditions.

The targeted number of visits and calls varied based on the content of the module. For the Safety and Ergonomics module, the research team targeted three in-person visits and three phone calls; for the Work Intensity module, the target was two visits and two calls; and for the Job Enrichment module, the target was two visits and three calls. The additional meetings for the Safety and Ergonomics module allowed for discussions of the assessment reports with the industrial hygienist who conducted the walk-through [53]. The technical assistance provided during these meetings offered an opportunity for the site manager and research team member to discuss progress, challenges, and concerns and to brainstorm potential solutions and available resources.

### Process evaluation

The implementation of the intervention was tracked by researchers at the site- and leadership-levels using quantitative and qualitative methods, thus allowing for triangulation of data across levels and methodologies in order to identify variations in intervention implementation.

Quantitative process tracking data monitored fidelity (the extent to which the intervention was delivered as planned) and dose (the amount of intervention delivered), following principles of process evaluation [54]. We tracked implementation of key intervention activities across the three sequential modules. At the site level, the research team member responsible for intervention delivery at each site documented implementation of intervention activities, including (1) completion of the assessment for each module, including a report of the findings and recommendations presented to the site manager; (2) discussion of plans for implementing the recommendations using the action planning approach; (3) in-person and phone meetings with the site managers to provide consultation and technical support to implement the recommended solutions to improving the targeted working conditions; (4) implementation of and participation in training and group activities to facilitate changes for two of the three modules; and (5) presentation and review of tools for engaging frontline workers in the targeted changes. We documented all intervention contacts, including date, length and method of contact (i.e., in person or by phone), planned conversation objectives and topics, and materials shared. Information was summarized by site, module, and type of intervention contact, and compared with the targeted intervention planned. The research team additionally documented planned intervention interactions that did not occur. The research team also tracked implementation of the intervention at the district and senior leadership level and documented participation, contributions and decisions based on meeting notes.

Qualitative data collected concurrent with and after intervention implementation used principles of realist evaluation [55] to assess the context of intervention implementation and explore barriers to and facilitators of intervention implementation and organizational change. Using qualitative data, we examined how the defined “essential elements” (Fig. 2) functioned as mechanisms of intervention implementation [55]. Qualitative data included information from the process tracking system recorded systematically by research team members, including their reflections on intervention delivery, such as observations on contextual factors and barriers to and facilitators of intervention implementation; and meeting notes and minutes from intervention-related meetings and communications to document the implementation of the intervention across the five sites and with the national and district leadership. Qualitative data also included post-intervention interviews conducted with intervention site managers (*n* = 5), intervention district managers (*n* = 2 interviews covering 3 sites), company national project champion (*n* = 1), operations manager (*n* = 1), human resources manager (*n* = 1), safety managers (*n* = 2) and a joint interview with the two research team interventionists (*n* = 2). Two of the district managers were each responsible for two of the five intervention sites. Post-intervention interviews were also conducted with eleven food service workers from one of the intervention sites.

Qualitative data from 25 post-intervention interviews and 89 process tracking documents were coded based on an inductive approach adapted for organizational research [56] and analyzed using thematic analysis [57, 58]. We used these analyses to identify themes regarding contexts and mechanisms (following our defined essential elements), based on the principles of realist evaluation [32]. Analyses of qualitative data were conducted in two rounds. In the first round, one member of the research team reviewed the interview and process tracking data and assigned codes to relevant sections of the documents using NVivo 12 (QSR International Pty Ltd.) [59]. This coding was then reviewed by a second member of the research team to ensure coding consistency and identify any differences in interpretation. In the second round, we grouped coded data from the transcripts and used process tracking field notes to triangulate and confirm the data from the interviews. Of note, neither study interventionist who was interviewed and developed the process-tracking documents was involved in the coding process. However, these study team members did participate in our final analysis discussions as a form of research team member checking [60].

## Results

### Workplace characteristics

Characteristics of the ten worksites participating at baseline are presented in Table 1. As shown here, one control site closed in the middle of the study and four additional sites were closed before data collection was completed due to COVID-19 related disruptions.

**Table 1:** Worksite characteristics

Worksites	Client Industry	Number of Frontline Workers	Account Stability^a^	Percent Completed Intervention Contact Points
Intervention Sites				
1	Law Firm	7	DM turnover (3) Chef turnover (2) No final data collection due to COVID-19	68%
2	Office Park	5^b^	DM turnover (2)	74%
3	Membership organization and Conference Center	10	Low turnover	63%
4	Banking	20	SM Turnover (4) DM turnover (2) No final data collection due to COVID-19	58%
5	Biopharmaceuticals	20		74%
Control Sites				
6	Biopharmaceuticals	7		
7	Analytical laboratory instrument and software company	10		
8	Military technology	11	SM Turnover (2) No final data collection due to COVID-19	
9	Medical School	17	Site closed mid-study	
10	Banking	17	SM Turnover (2) No final data collection due to COVID-19	

^a^ *SM* Site Manager; *DM* District Manager (DM turnover not tracked in control sites)

^b^ Met eligibility requirements with additional temporary employees

### Intervention implementation: results from quantitative process tracking data

The intervention was implemented at five sites with support from district managers and senior leadership, focusing on the implementation of three modules that targeted identified priority working conditions. We analyzed our process tracking data to assess the extent to which the intervention was implemented as planned and the dose of intervention delivered based on number of contact points (i.e., in-person visits or meetings, phone calls).

#### Site level intervention

A member of the research team conducted an assessment related to each module as the basis for recommendations and an action planning process. The assessments were conducted and reports presented to site managers of the five intervention sites for each of the three modules, with the exception of one site, which did not receive a report for the Job Enrichment module due to turnover of the site manager.

The intervention plan specified that the research team would work with site managers to develop an action plan based on the report for each working condition. The action planning tool, adapted from a tool already in use by the company, was intended to offer a structure for outlining tasks to address the identified problem, a timeline for task completion, and a strategy for assigning task completion to specific employees. Based on feedback from site managers after the first module, the research team revised the action planning tool for simplicity and ease of use. Nonetheless, no site manager completed the action planning tool for any of the modules, citing lack of time and job demands as barriers. Instead, the research team member reviewed the action planning process and tool with each site manager and used site-specific examples to illustrate opportunities for improvement for each module. Thus, while the action planning tool itself was not utilized, the concepts outlined provided an approach to address priorities identified through the assessments and develop plans for making changes during the in-person and phone consultations with the research team.

The targeted number of visits and calls varied based on the content of each module. As shown in Table 2, the contact points were lowest for the Job Enrichment module, which also occurred during a busy holiday time period that presented significant competing priorities. In addition, three group sessions were conducted for all site managers with the goal of coordinating implementation and communications across intervention sites. Overall, the research team implemented approximately two-thirds of the intended contacts with site managers.

**Table 2:** Worksite-level Intervention: Mean number of contact points with Site Managers across the five intervention sites, by module and type of contact

Module	In-person Visits	Phone Calls	Group training/ discussion	Total contact points/planned
Project introduction and cross-site coordination	1/1	NA	1/1	2/2 (100%)
Safety & Ergonomics	2/3	1.8/3	NA	3.8/6 (63%)
Work Intensity	1/2	1.8/2	1/1	3.8/5 (76%)
Job Enrichment	.2/2	2/3	.8/1	3/6 (50%)
Total contact points	4.2/8 (53%)	5.6/8 (70%)	2.8/3 (93%)	12.6/19 (66%)

The research team offered to attend the huddles with frontline workers to address issues raised for each of the working conditions with the intention to encourage discussion and action planning that would involve both the site manager and the employees. Research team members attended huddles for four of the five sites for the Safety and Ergonomics module, but were not invited to attend huddles for the other two modules due to the sites’ time constraints and competing demands on site managers and frontline workers.

#### Leadership intervention

The research team met with senior leadership and district manages throughout the intervention, as summarized in Table 3. Rather than targeting a predetermined number of contacts, the number and type of contacts with leadership representatives was determined based on needed input for each module.

**Table 3:** Leadership-level Intervention: Contact Points with Leadership Representatives

Module(s)	Contact Points	Participants	Objectives
Project Introduction and Module Coordination	Two in-person meetings	Senior VPs representing Health and Safety, Human Resources and Operations	Introduce study; co-develop a shared vision for the intervention; identify potential internal resources
Safety and Ergonomics, Work Intensity, Job Enrichment	Four telephone meetings	District Managers	Quarterly meetings for reviewing module assessment reports, identifying internal resources for addressing site level priorities, supporting Site Manager participation
Safety and Ergonomics (S&E)	Two in-person meetings Two telephone meetings	Health and Safety leadership	Provide input on the development of the S&E walkthrough assessment; align priorities with ongoing internal safety audits; review aggregate walkthrough findings; identify existing company resources and opportunities for addressing areas identified for improvement
Work Intensity (WI)	Four telephone meetings	Human Resources and Operations leadership	Review and discuss policies, practices and resources; explore challenges and opportunities to address WI across sites
Job Enrichment (JE)	Five telephone meetings One in-person meeting	Human Resources and Operations leadership	Review available tools and resources and consider adaptations for use with frontline workers; identify opportunities to highlight performance goals and career advancement

### Context and process: results from qualitative data

To understand factors influencing intervention implementation, we analyzed qualitative data to explicate the extent to which the organizational context hindered or facilitated the process. Barriers within the work setting reflected the nature of food service in general, the complexities of the relationships between the parent employer and the client/host company, and competing priorities within the specific parent employer. We adapted the intervention in response to the identified challenges.

This setting illustrates the challenges of a complex system with various interacting elements, including the parent employer, the host organizations and the frontline workers. The environment was generally characterized by low profitability, low wages, high turnover, conflicting demands, and limited potential to modify the workspace because of the contracting relationship. Our interviews underscored that scheduling was complicated; frontline workers often worked multiple jobs to make ends meet and some needed to balance work with childcare arrangements. Frontline workers’ low wages contributed to high turnover. This food service company was competing for skilled staff; other employers, like hotels and restaurants, could afford to pay more. During the intervention, turnover presented a significant barrier across multiple levels, including among district managers, site managers, and frontline workers, with implications for continuity and engagement at the site:

*“One that we systemically deal with is turnover … , from the district level down to the frontline supervisors. So continuity – I mean, if you’re gonna implement changes, you have to have some continuity. Someone supports the change and manages it and implements it and briefs the employees – that’s part of it.” (District Manager).*

*“The other piece of it – 14 months in our industry is an eternity. So when I looked at the accounts that were in the pilot, there was a lot of turnover in those positions. So looking at this list [of participating sites and site managers], he’s gone – he’s gone. He found another opportunity. That’s part of the lifecycle of our organization, and in this industry there’s turnover. So that’s been a bit of a challenge.” (Operations Manager).*

The challenges in the work environment were further complicated by relationships with host organizations. As illustrated in the quotes below, responding to client requests was a top priority; and because relationships with clients were a top priority, site managers were often unavailable to participate fully in the intervention:

*“The relationship [is] with the client, so if it’s volatile or shaky... we don’t want to do anything except please them and stick to our core business which is executing food. Right? So anything perceived as taking away time from that is not helpful.” (National Project Champion).*

*“Work intensity in particular was interesting … . it’s our mission to satisfy the customer, the client, [so we] take last-minute requests and catering orders. And that naturally adds to the work intensity. So, I think it was just very eye opening, that that was a struggle, and I don’t think we came up with a solution though, necessarily. Because the easiest solution would be, well, just stop taking these last-minute requests or re-educate the customer, but, easier said than done.” (District Manager).*

The corporate client was the gatekeeper for some of the resources needed for making recommended changes. For example, the client designed and provided the physical space for the food services, and accordingly, could set limits on potential improvements. The following quote from a site manager in response to recommended changes illustrates the implications for the Safety and Ergonomics module:

*“There are some that the client won’t allow to change. Specifically, adding a chair and a mat for the cashier. For aesthetics, the client won’t allow this. The account has no choice. Salad bar is old and needs updating or to be replaced. Client is responsible for maintenance … It’s their equipment. Most of the items on the list require client involvement and expenditures since the account doesn’t own the equipment – the client does.” (Site Manager).*

The potential to lose a client contract was an ongoing source of anxiety about job security for site managers. Low profit margins in the food service industry in general dictated the need for efficiencies and productivity. For this parent company, financial constraints led to increasing cost-cutting measures and mounting budgetary pressures, illustrated in this quote:

*“There used to be more floating managers and floating chefs so that if a chef or a manager had to call out sick or they wanted to take a week off, they were able to because there were resources to step in and cover for them. And that dwindled and then it disappeared because of financial constraints. Similarly, when they got new registers, it used to be that they had IT technology support to come in and program those registers. Now that’s something that’s being asked of the [site] manager.” (Research Team Member).*

Frontline workers discussed how these pressures and the need for efficiency and productivity influenced their day-to-day demands:

*“The food business is very demanding. It’s very unique. And I think a lot of people – from the outside looking in, you might not realize how intense of a business it is. I mean, that’s something I don’t think – if someone says, oh, you’re a chef. They think, oh, you make food all day long. And it’s like, you don’t know how many deadlines I hit all day long. It’s like you have a deadline to open the restaurant at this time, you have a deadline for every catering [job], you have a deadline with every vendor, and all – lots of things are coming at you at the same time, especially in a place like this that does a lot of catering.” (Frontline Worker).*

Restructuring and downsizing across the organization also played a role, with changes in leadership engaged in the project and new priorities superseding the intervention. Site and district managers also reported new and ongoing competing demands on their time, including the implementation of a new register system, new reporting requirements, changes in catering demands, contract restructuring, and ongoing financial demands.

The organizational changes targeted by the intervention represented a culture change that may have required greater organizational investment than was available across all levels of the organization. As illustrated by the following quote, this need for a culture change was prioritized at the national level:

*“I look at priorities as things that change all the time … It’s taken from a priority to more of a culture shift …*. *It’s trying to get that culture shift in the organization. And we’re starting to see that shift turning into the direction which is good, which will ultimately benefit that frontline employee where they’re doing the work and putting their health and well-being at risk on a daily basis with the jobs that they do.” (National Project Champion).*

Organizational change, however, requires significant commitment across various levels of leadership within the organization. Although senior leadership initially communicated support for the program, site managers reported that support was not communicated or sustained:

*“So there wasn’t a lot of assistance above me for doing this. It was brought to me, I took that direction and then at one – the only time we had a general meeting of everyone at [research team location] we discussed some issues, and it never really went much further than that. So to say that there was a lot of assistance from those above us, there was a bit right after the meeting, but then that’s kind of where it ended.” (Site Manager).*

Indeed, some leadership representatives reported that they saw their role in supporting the intervention as simply staying informed, rather than communicating about or encouraging engagement in the intervention, as illustrated here: ***“****I was informed … So my role wasn’t to communicate [about the pilot] to anyone else.” (Safety Leadership).*

District managers, who directly supervised site managers, placed a high priority on financial outcomes and deliverables. As a result, site managers reported that they generally did not feel comfortable sharing needs and ideas with district managers. When asked about communication with the district manager, one site manager reported it was:

*“Terrible. Terrible. There isn’t any [communication] …*. *We won’t hear from the district manager unless there’s something wrong …. They work a lot with the clients to make sure the clients are happy, looking at the financials. So that’s kind of it … I mean the accounts really sort of felt like they were on their own and that the upper levels had no clue about what it took for them to manage the accounts. So the communication, I would say, is terrible.” (Site Manager).*

As a consequence, the research team often served as the primary conduit for communications related to the intervention across levels of the organization, as noted by one district manager: *“All of our interaction has been with you folks.”*

The content of the intervention was informed by our formative research [43] and focused on the three working conditions targeted by the intervention modules. Leadership reported that these targeted working conditions were a good fit in that they aligned with existing company priorities:

*“Job enrichment, I think just the fact of, again, bringing awareness to it. That’s something we’re trying to do with our employees, whether it’s through cross-training and getting more aware of different job responsibilities, or in some cases – I don’t think we got into it necessarily, but with education opportunities for us, for our staff and ability for them to grow. But I think, again, it’s just two really good concepts that we don’t spend enough time on, that the study brought awareness to.” (District Manager).*

*“Well, if you talk about environment, safety, employee engagement, I mean I think it’s safe to say it’s pretty well aligned with our strategic priorities as an organization, right? I would say it hits really the key pillars of what we are trying to accomplish with our associates in the workplace.” (Operations Manager).*

Nonetheless, despite the strong fit of the content of the intervention with the stated corporate priorities, site managers pointed out challenges that reduced fit, including the significant competing priorities and time demands that limited site managers’ time and ability to participate in the intervention. Workload pressures were a significant concern for site managers, who as salaried employees, often needed to complete work tasks outside their standard work hours. Site managers reported difficulty taking vacation and many covered for chefs and other staff members who were taking time off:

*“It’s so busy for the most part that it’s just finding the time to implement those things, and it’s always been a challenge for us. And it doesn’t make your study any different than our normal checks and balances. It’s just the fact of it is that being able to sit down and make time with everybody and – it’s never been easy.” (Site Manager).*

The research team made adaptations throughout the intervention to improve fit. For example, although the intervention was initially planned for greater participation of frontline workers in the implementation process, based on input from site managers the research team adapted the approach to focus on site managers as the primary gatekeepers within the worksites:

*“We are the ones that set the tone and pass through all the information. We know how to do that better than anybody because we work directly with all the different personalities that are here … it was handled correctly, in my eyes.” (Site Manager).*

Plans for engaging frontline workers included huddles, which were an existing regular communication vehicle for site managers to engage workers, as well as through one-on-one conversations and existing committees, such as health and safety committees. Research team members observed that frontline workers attended huddles but rarely spoke. However, research team representatives were offered few opportunities to participate in huddles or committee meetings.

*“We planned to have a short huddle after our meeting. When the [Site Manager] went back to the kitchen, he then came back out and told me that they were behind and couldn’t take time away from prep work to participate in a huddle.” (Research Team Member).*

## Discussion

The Workplace Organizational Health Study tested the implementation of an organizational intervention to promote and protect the safety, health, and well-being of low-wage food service workers employed in contracted cafeterias. Focusing on five sites randomly assigned to the intervention, we examined two key questions: (1) to what extent was the intervention implemented as planned (fidelity) and what was the “dose” of intervention implemented; and (2) to what extent did the organizational and broader contexts hinder or facilitate the process of implementing the intervention? Over the 13-month intervention, research team representatives met approximately monthly with site managers, delivering consultation and technical assistance on each of the three modules and implementing approximately two-thirds of the intended contact points. In addition, the intervention implementation included collaboration with a multi-level leadership team, which was engaged to provide resources and support for the intervention. The financial pressures, competing priorities and fast-paced work environment placed constraints on site managers’ availability and limited their engagement in the intervention. This study benefited from a rigorous process tracking framework and carefully analyzed quantitative and qualitative data to shed light on the implementation process. By assessing the context of the food service setting in which the intervention was situated, we were able to explore factors hindering and facilitating the implementation of the intervention.

### Implementation mechanisms and process

We analyzed qualitative data from the process tracking and post-intervention interviews to understand the role of four essential elements we considered mechanisms in the process of intervention implementation. *Leadership support and commitment* is necessary to prioritize worker safety and health, ensure availability of resources, and reinforce the need for accountability. Senior leaders voiced support for the intervention and committed to the intervention at its start. For example, health and safety leaders contributed to the development and implementation of assessments and reports, and linked findings to resources provided by their team. Senior leaders also identified company tools to apply to the intervention, such as for providing coaching and feedback as part of the Job Enrichment module. However, support from the district leadership was impeded by turnover, competing priorities, and lack of resources. Our results are in line with previous research that has found that leadership can make or break an intervention [61]*. Communication* is necessary to facilitate effective collaborative relationships, both vertically and horizontally [62]. Our research pointed to communication barriers between organizational units, with lack of communication common between site managers and the district leaders to whom they reported. Previous research has also found that lack of communication among leadership levels may have a detrimental effect on the intervention process [63]. Using a *participatory approach,* the intervention was designed to engage key stakeholders across levels of the organization, including district-level managers, site managers, and frontline workers. Despite initial plans for engaging frontline workers, the research team found few opportunities for their direct involvement, likely reflecting time constraints and usual patterns of communications. Frontline workers faced the time demands and pressures of this setting, along with potential complications with balancing work with family needs and often with a second job. A contribution of this study is that it highlights important questions about best practices for implementing participatory interventions in high intensity work settings. *Tailoring for fit* reflects the need to customize the intervention to the organizational context [64]. Findings from formative research guided selection of the three targeted working conditions, seen as significant priorities across all organizational levels. The research team further tailored the intervention to fit each worksite based on assessments specific to each site and module, using these findings to provide consultation and technical assistance to site managers. Although site managers were reluctant to use a formal action planning process, the research team adapted the approach as part of the consultation process. Others have similarly recommended that interventions cannot be implemented in a linear pattern but require iterative adjustments to maximize fit [65].

### Context

This intervention was shaped by multiple contextual layers, including the inherent challenges of implementing an organizational intervention in the context of a research study, the nested relationships of the worksites within the broader hierarchical setting, and the complexity of relationships between the employer organization and their contractual clients.

Conducting an organizational intervention in the context of a research study introduces constraints that may not be present when changes are implemented from within the organization. In the research context, following a standardized protocol ensures that the study is testing a defined intervention, as illustrated by the three intervention modules, based on specific working conditions identified through formative research [43], that structured this intervention. To the extent possible, we also tailored the intervention to each site and adapted our approach as we learned more about the context, demands, and constraints. We recognized that the recommended organizational changes required significant support from senior leadership and an investment of resources to ensure success. As outsiders, we were able to work with site managers and leadership to identify probable risks to workers and generate potential solutions; ultimately, however, decisions about the objectives to be tackled and the resources to be made available rested with corporate leadership and reflected the organizational culture and demands. For example, despite prioritizing work intensity as a target for this organizational intervention, modifications in staffing patterns or workflow were not considered as possible solutions. Decision-making authority rested with varying levels across the organization. Although senior leadership expressed their support for the study and the intervention, we were not privy to internal conversations that may have shaped implementation of the intervention. In addition, contracts with the client may have required completion of work with additional costs, thereby imposing budget limitations that restricted options for diminished work intensification. Existing organizational norms and perceived resources may have shaped the business value placed on workforce safety, health and well-being. Investment in the cultural changes represented by this intervention may have been a stretch in light of more immediate pressing demands and priorities. The aspirational goals reflected by some representatives of national leadership ultimately met with on-the-ground realities due to resource constraints.

The nature of the contractual relationships with the hosts for these food services likely played a significant role in the implementation of this intervention. The implications of the fissured workplace—with its blurred lines of accountability among multiple employers – have received increasing attention in discussions about worker safety and health [26, 66]. These challenges have been documented in other industries, such as construction [67, 68], but little research has focused on food service. In our study in the food service industry, we found that as a result of contractual obligations to the client organization, site and district managers prioritized clients’ needs, often at the expense of programs and practices that benefit employees but do not generate revenue. We also encountered obstacles to changing the conditions of work, such as the physical environment, that were owned and managed by the client.

For organizational interventions in contracted settings, it may be necessary for researchers, intervention implementers, and policy planners to consider this dual responsibility for the work environment. In this study, although the client companies were informed of the study, they were not actively engaged in the process of intervention planning and implementation. In many countries, such as the U.S., [69] there is a joint responsibility between the host and the employee companies to protect workers’ safety, health, and well-being. Further, strategies that engage both the client as well as the employing company to work collaboratively are likely to be more effective. These collaborative initiatives ensure support and commitment from both companies for the project, and potentially enable the intervention to be implemented further upstream. We observed that specific conditions covered by the contracts (e.g., equipment, hours of work, responsibility for catering) varied by site, with each site covered by site-specific contracts. Interventions in similar settings might consider recommending that the parent employer include in contract language permissions for employers to implement changes in the work environment as well as the costs for implementing such improvements. Similarly, future health and safety legislation might reflect the dual responsibilities for workers’ health and safety of both parent employers and host clients.

### Strengths and limitations

The strengths of this study include its focus on an organizational intervention in a low-wage work setting, the mixed method process evaluation capturing the extent of intervention implementation and the barriers and facilitators to implementation, and its exploration of these factors in a fissured work environment. This study also faced several limitations in its implementation. Foremost among these was the timing of the final data collection, which was not completed due to shutdowns related to the COVID-19 pandemic. Due to incomplete final data collection, we were unable to analyze the process evaluation data alongside the quantitative health and well-being outcomes and were unable to report quantitative changes in working conditions. Lacking the ability to collect final outcome data limited the conclusions we wee able to draw from this study, necessitating instead that we focus primarily on process data rather than on the effects of the intervention on changes in working conditions and worker safety, health and well-being, as intended. We also recognize that sites were not equally represented in the final qualitative data collection due to site closures and worker lay-offs; food service workers participating in qualitative data collection represented only one site. However, although not ideal, sites not included in the food service worker focus groups were represented in the leadership and the interventionist interviews. Instability in sites and personnel turnover further complicated the study; one worksite closed prior to COVID-related shutdowns, and there was significant turnover among site managers, district managers, and senior leaders during the 13-month intervention period. Further, there is a potential for recall bias; because the intervention was implemented over a 13-month period, those stakeholders interviewed may have recalled the more recent components of the intervention and barriers to implementation, rather than those that occurred earlier in the intervention. Although we could never have forseen the impact of the COVID-19 pandemic on this study and its follow-up data collection, future studies may benefit from collecting interim qualitative data collection with key stakeholders, such as workers and middle managers. In this study, it would have been beneficial to conduct, for example, interviews after each of the modules.

We additionally recognize that the experience of implementing the intervention from within the organization may have yielded different results and processes from those offered here from the perspective of external researchers. Although additional contact points might have strengthened the effect of the intervention, in this context further contacts in implementing the intervention were not feasible in light of competing organizational priorities and constraints; basically, the implemented intervention saturated what was possible within this organization.

## Conclusions

Working conditions, such as physical exposures, job demands, or psychosocial experiences, not only shape injury and illness risk, but may also may provide avenues toward improved well-being. This study contributes to an evolving literature on the role of the work organization in determining worker health outcomes, part of the growing effort to understand how work may serve as an important social determinant of health [70].

This organizational intervention study illustrates the application of a well-articulated conceptual framework for promoting improvements in three working conditions (safety and ergonomics, work intensity, and job enrichment), with attention to four essential elements (leadership commitment, communication, participation, and tailoring for fit) seen as mechanisms of the change process. We tailored the intervention to the setting using an iterative process as we identified barriers and facilitators of change and improved our understanding of the multi-layered contextual setting. Despite strong initial support from the parent employer’s senior leadership, we encountered barriers in the implementation of the intervention at the worksite and district levels, reflecting fiscal demands that drove work intensity; turnover of site and district managers that contributed to lack of continuity in the implementation of the intervention; and time demands and staffing constraints that further increased workload and pace for managers and frontline workers alike. This study also underscored the significance of contractual relationships with client/host organizations, which were often central decision makers around changes to the work environment and may need to be involved from the inception of planning for such interventions. Despite these challenges, research staff were able to provide consultation and technical assistance in monthly meetings with site managers, implementing two-thirds of the intended contacts with site managers by phone and in person, and to engage district and senior leaders in intervention planning and identifying resources to support its implementation. Due to the COVID-19 shutdowns, final data collection from frontline workers and some site managers was curtailed, and as a consequence, findings on potential improvements in working conditions and workers’ safety, health and well-being are not available. Nonetheless, an important contribution of this study is found in the process evaluation of intervention implementation, thereby providing an intermediate indicator of impact.

This study points to several important recommendations. These results underscore the importance of the work context in planning for and implementing organizational interventions. We observed a hierarchical process in which site managers reported to district managers, were dependent on senior leadership for corporate resources, and additionally were constrained by contracts with client companies that hosted the work environment of employees. Although we worked closely with site managers responsible for worksite operations, we found that many organizational changes required resources and support from decision makers at other levels. These findings highlight the need for identifying the key gatekeepers and decision makers for targeted organizational changes. Future research will also benefit from considering the increasing complexity of work relationships, especially in fissured work settings. The contributions of this study include an expanded understanding of the process of implementing an organizational intervention in a low-wage food service setting; consideration of the complexities introduced by the fissured work environment, with blurred accountability for worker health and safety; and insights into the barriers to and facilitators of the process of implementing this organizational intervention in this complex setting.

## Data Availability

The datasets generated and/or analyzed during the current study are not publicly available due to privacy restrictions with the collaborating employer but may be made available from the corresponding author on reasonable request.
